# Chitosan Nanoparticles Rescue Rotenone-Mediated Cell Death

**DOI:** 10.3390/ma12071176

**Published:** 2019-04-11

**Authors:** Jyoti Ahlawat, Eva M. Deemer, Mahesh Narayan

**Affiliations:** 1 Department of Chemistry & Biochemistry, The University of Texas at El Paso, El Paso, TX 79968, USA; jahlawat@miners.utep.edu; 2 Material Science & Engineering department, The University of Texas at El Paso, El Paso, TX 79968, USA; emdeemer@utep.edu

**Keywords:** chitosan (CS), anti-oxidant, anti-apoptotic activity, rotenone, Parkinson’s disease (PD)

## Abstract

The aim of the present investigation was to study the anti-oxidant effect of chitosan nanoparticles on a human SH-SY5Y neuroblastoma cell line using a rotenone model to generate reactive oxygen species. Chitosan nanoparticles were synthesized using an ionotropic gelation method. The obtained nanoparticles were characterized using various analytical techniques such as Dynamic Light Scattering, Scanning Electron Microscopy, Transmission Electron Microscopy, Fourier Transmission Infrared spectroscopy and Atomic Force Microscopy. Incubation of SH-SY5Y cells with 50 µM rotenone resulted in 35–50% cell death within 24 h of incubation time. Annexin V/Propidium iodide dual staining verified that the majority of neuronal cell death occurred via the apoptotic pathway. The incubation of cells with chitosan nanoparticles reduced rotenone-initiated cytotoxicity and apoptotic cell death. Given that rotenone insult to cells causes oxidative stress, our results suggest that Chitosan nanoparticles have antioxidant and anti-apoptotic properties. Chitosan can not only serve as a novel therapeutic drug in the near future but also as a carrier for combo-therapy.

## 1. Introduction

Parkinson’s disease (PD) is a multifocal progressive neurodegenerative disorder clinically defined by the presence of akinesia, postural instability, muscular rigidity, and tremor [1]. It is the second most common neurodegenerative disease and is prevalent in 0.1–0.3% population with an increased frequency observed in patients ≥65 years [2]. Interestingly, PD patients often display non-motor signs and symptoms such as sleep disturbances, mood deflection, anosmia, gastrointestinal dysfunction (e.g., 80% of patients suffer from constipation), sexual-urinary dysfunction, thermoregulation changes, neuropsychiatric problems, cardiovascular disturbances, and pain [1,3]. PD is characterized by the selective loss of dopaminergic neurons in the substantia nigra pars compacta and arises due to the deposition of insoluble polymers of α-synuclein in the neurons, forming spherical intracytoplasmic inclusions known as Lewy bodies [1]. These lamellated cytoplasmic bodies eventually result in neurodegeneration and the death of dopaminergic neurons [1]. This neuronal death is associated with disruption in cellular hemostasis, resulting in disruption of the nuclear membrane integrity, signaling α-synuclein aggregation which later propagates to other neurons by direct or indirect means [4]. Furthermore, studies have shown that α-synuclein aggregation impairs axonal transport and exerts a detrimental effect on the health of neurons due to the activation of neighboring inflammatory microglial cells [3].

Parkinson’s disease can be either inherited or sporadic in nature. Although, familial PD accounts for around 10% of the cases, sporadic PD has been found to account for the remaining ones. Moreover, the etiology of PD is not completely understood but sporadic PD is believed to originate from interaction of individual genetic susceptibility and environmental exposure [5,6]. Probably, there is not one single factor that is solely responsible for causing the disease. Rather, there exists several factors acting simultaneously [7]. Previous studies have suggested that pesticides such as rotenone are involved in the increased risk of Parkinson’s disease [5]. In addition to being a pesticide, it is a potent, highly specific inhibitor of complex I of the mitochondrial electron transport chain (ETC) [6]. Unlike N-methyl-4-phenyl-1,2,3,6-tetrahydropyridine (MPTP), which causes defects in complex I of ETC in catecholaminergic neurons, rotenone causes complex I inhibition, uniformly, across the brain [8]. Its hydrophobic structure allows easy penetration through the blood–brain barrier and cell membrane. The selective toxicity of this lipophilic compound is relevant because of its wide usage as a herbicide in gardens and as a delousing agent for animals and humans [7]. Furthermore, studies have shown degradation of selective nigrostriatal dopaminergic neurons upon rotenone infusion, reproducing pathological features of clinical Parkinson’s disease [6]. Moreover, a study by Niyanyu et al. showed that rotenone can induce mitochondrial reactive oxygen species (ROS) production which is closely related to rotenone-induced apoptosis [9]. 

Since no viable treatment exists for PD, there is an urgent and unmet need for the development of novel therapeutic agents to either cease or reverse the symptoms or the progression of this progressive age-related disorder [10]. Synthetic compounds are associated with various side-effects [11]. Therefore, there is a need to find some natural neuroprotective agent that has the ability to scavenge ROS and hence defer the progression of Parkinson’s disease [10]. Chitosan is a cationic polysaccharide, composed of a linear chain of D-glucosamine and N-acetyl-D-glucosamine linked via a β (1,4) bond, obtained from an alkaline N-deacetylation of chitin [12]. This marine shrimp-derived carbohydrate possesses well-documented antioxidant properties with minimal or no side-effects observed. Moreover, they also exhibit neuroprotective, anti-hemorrhagic, anti-tumor, anti-diabetic, anti-viral, and antibacterial effects. Furthermore, it has mucoadhesive properties allowing easy penetration of this carbohydrate through the well-organized epithelia [13]. In a study, in 2001, Gilgun-Sherki et al. showed that elevated ROS production and an imbalance between pro-oxidant and antioxidant activity (e.g., superoxide dismutase, catalase, and glutathione peroxidase enzyme) leads to neuronal death and hence a diseased condition [14]. In a different study, Guo et al. in 2006 reported that fucoidan (sulfated polysaccharide) could reverse changes such as superoxide dismutase activity and alleviate the reactive oxygen species level in PC 12 cells when exposed to hydrogen peroxide [15]. Later, Gao et al. (2012) showed the antioxidant effect of fucoidan on hydrogen-peroxide-treated PC12 cells and the pathway associated with it [16]. In a different study, Xie et al. (2014) reported antioxidants could alleviate the reactive oxygen species level [17]. Further, in 2016, Wang et al. reported fucoidan pretreatment could rescue the cells from oxidative stress, protein carbonyl lipid peroxidation, and mitochondrial dysfunction [18,19]. Related to this, Liu et al. (2016) showed the effect of sulfonated chitosan on the differentiation of neuronal cells and exhibited immunomodulatory effects [20]. Recently, Magnigandan et al. (2018) reported the anti-oxidant and ROS scavenging activity of low molecular weight sulphonated chitosan, where they found that rotenone insult resulted in antioxidant depletion and lipid oxidation causing cellular damage, oxidative stress, mitochondrial dysfunction, and hence, a diseased state which were reversed by the action of low molecular weight sulfated chitosan [11]. 

However, there are no reports on the in vitro neuroprotective effects of bare chitosan nanoparticles. Therefore, the goal of this study was to exploit the antioxidant and anti-apoptotic activity of the prepared chitosan nanoparticles for evaluation in vitro, against a human SH-SY5Y neuroblastoma cell line. Hence, rotenone was used as the causative agent for inducing PD in the SH-SY5Y cell line and then, the therapeutic neuroprotective efficacy of the synthesized chitosan nanoparticles was evaluated. Therefore, we propose that chitosan nanoparticles might be a potential candidate for the prevention of PD. 

## 2. Materials and Methods 

### 2.1. Materials

Chitosan (>75% deacetylated), and Sodium tripolyphosphate (Na-TPP) were purchased from Sigma-Aldrich (Saint Louis, MO, USA). Whereas, Dimethyl sulfoxide and acetic acid were ordered from Fisher Chemical (Hampton, NH, USA).

### 2.2. Preparation of Chitosan Nanoparticles Using Ionotropic Gelation Method 

Chitosan nanoparticles were synthesized using the Calvo et al. 1997 method [21]. In this, 0.175% (w/v) chitosan powder was dissolved in 1% (v/v) acetic acid and kept on a magnetic stirrer for overnight stirring at a temperature between 25–28 °C. Later, the pH of the solution was adjusted to 5.2 using 1M NaOH followed by addition of 0.1% (w/v) sodium tripolyphosphate in a dropwise fashion. The chitosan solution was then stirred at 1000 rpm for 10 min. The solution was centrifuged at 20,000 rpm (Sorvall RC-5B refrigerated centrifuge, Fisher Scientific, Hampton, NH, USA) for 90 min at a temperature of 4 °C to pelletize the chitosan nanoparticles. After centrifugation, the supernatant was discarded and the pellet was washed with deionized water three times. Ultrasonication was performed using a probe sonicator (Branson Sonifier 450, Emerson Electric Company, St. Louis, MO, USA) in an ice bath for 10 min at an amplitude of 30%. The obtained suspension was then immediately freeze dried using lyophilizer (Labconco, Kansas City, MO, USA). After freeze-drying, the samples were stored at 4 °C to carry out further analysis. 

### 2.3. Characterization of Nanoparticles

#### 2.3.1. Determination of Average Size, Polydispersity Index, and Zeta Potential Using Dynamic Light Scattering

The average size, polydispersity index, and surface charge of the nanoparticles was determined using DLS (Dynamic light scattering) at 25 °C. A total of 1 mL CS NP solution was diluted ten times in deionized water and the sample was analyzed using a Malvern Zetasizer ZS90 (Malvern Panalytical Ltd., Malvern, UK). 

#### 2.3.2. Scanning Electron Microscopy (SEM) 

The surface morphology of the prepared chitosan nanoparticles was analyzed using the S-3400N Type II scanning electron microscope, Pleasanton, USA (Hitachi High-Technologies Corporation, Tokyo, Japan). The liquid samples on a glass slide were dried overnight in a sterilized fume hood and later, the glass slide was mounted on a stainless stub using double-sided carbon tape. The sample was then coated with gold using a gold sputterer for 30 s to make the sample conductive. The images were recorded at an accelerating voltage of 2 kV and probe current below 20 µA. 

#### 2.3.3. Transmission Electron Microscopy (TEM)

The morphology of the nanoparticles was studied using the H-7650 transmission electron microscope, manufactured by Hitachi High-Technologies Corporation in Pleasanton, CA, USA. A single drop of the CS Nanoparticle dispersion was placed on carbon 400 mesh copper grid (Ted Pella, Redding, CA, USA). The copper grids were then dried overnight in a sterile fume hood and the images were taken using the Quartz PCI version 8 software (Quartz Imaging Company, Vancouver, BC, Canada) in TEM mode (200 kV). 

#### 2.3.4. Atomic Force Microscopy (AFM)

The surface roughness and morphology of the chitosan nanoparticles was further investigated using AFM (NT-MDT NTEGRA) in non-contact mode using Ted Pella TAP 150AL-G tip (Redding, CA, USA) with a radius of <10 nm. After capturing the images, the data analysis was performed using NOVA software (NOVA Company, ChongQing, China).

#### 2.3.5. Fourier Transform Infrared Spectroscopy (FTIR)

The IR spectra of the Chitosan nanoparticle sample was obtained using a Nicolet, Thermo Scientific FTIR instrument (Waltham, MA USA). The sample was ground to fine powder along with a KBr pellet. The scanning range was from 500–4000 cm^−1^. The data were analyzed using OMNIC software (Fisher Scientific, Hampton, NH, USA). 

### 2.4. Cellular Behavior of Human SH-SY5Y Neuroblastoma Cell Lines on Treatment with Samples

#### 2.4.1. Cell Culture

Annexin V- FITC apoptosis kit (Beckman Coutler, Brea, CA, USA), Hoechst 33342 fluorescent satin (Invitrogen, Carlsbad, CA, USA), propidium iodide (PI) (Invitrogen, Eugene, OR, USA), Fetal Bovine Serum (FBS) (Atlanta Biologicals, Atlanta, GA, USA), and a human neuroblastoma cell line SH-SY5Y (ATCC, Manassas, VA, USA) were purchased. SH-SY5Y cells were cultured in DMEM and Hans’s F12 media mixture (1:1) comprising of 10% FBS (v/v) supplemented with 1% (v/v) penicillin-streptomycin and maintained at 37 °C in an incubator with 5% CO2 atmosphere. Cells were sub-cultured every 48 h and Trypsin-EDTA 0.25% (1×) was used to detach cells from the culture surface when needed.

#### 2.4.2. Differential Nuclear Staining Cytotoxicity Assay

The cytotoxicity of different concentrations of chitosan nanoparticles, chitosan powder, and rotenone were evaluated. Cells were first cultured on 96-well plates and incubated for 24 h to allow attachment to the culture surface. Later, cells were treated with a different concentration of rotenone and chitosan nanoparticles to determine the possible cytotoxic effect of the added treatments. Subsequently, untreated cells were taken as a negative control and hydrogen-peroxide-treated cells were taken as a positive control. Moreover, to determine the cytotoxic effect of rotenone on the cells, the cells were treated with chitosan nanoparticles (different concentrations) 6 h prior to rotenone exposure. Subsequently, cells were further incubated for 24 h. Later, 1 µg/mL mixture of PI/Hoechst 33342 was added to each well in the 96-well plates 1 h prior to the imaging process [22]. The images were captured using a Bioimager system (BD Biosciences Rockville, Montgomery, MD, USA). Five images were taken per well using a 10× objective lens. Subsequently, BD AttoVision v1.6.2 software (BD Biosciences Rockville, Montgomery, MD, USA) was used to determine the percentage cell death per well.

#### 2.4.3. Flow Cytometric Assay 

The SH-SY5Y cell lines were seeded at a density of 20,000 cells per well in a 24-well plate and incubated for 24 h to allow attachment of the cells to the culture surface. Cells were then incubated with various concentrations of chitosan nanoparticles prior to rotenone exposure and subsequently, cells were incubated for an additional 24 h. Cells from each well were then collected, washed, and processed [23]. Briefly, cells were concurrently stained by suspending them in a solution containing annexin V-FITC (PI) in 100 µL of binding buffer (Beckman Coulter, Brea, CA, USA). After incubation for a time interval of 15 min on ice, in a sterilized Lab Safety Cabinet II in dark, 400 µL of ice-cold binding buffer was added to the cells. The resulting suspension was then homogenized gently and subsequently, analyzed using a Cytomics FC 500 Beckman Coulter Flow cytometer. For each sample, approximately 10,000 events were captured and data analysis was performed using Beckman Coulter CXP software. 

#### 2.4.4. Mitochondrial Membrane Potential (ΔΨm)

The loss of mitochondrial membrane potential is a hallmark for cellular apoptosis. Briefly, SH-SY5Y cells were seeded onto a 96-well plate for 12 h. Subsequently, cells were treated with chitosan nanoparticles 6h prior to rotenone treatment. Later, after 24 h of incubation, the cells were incubated with rhodamine 123 dye at 37 °C for 30 min. Finally, the mitochondrial membrane potential was evaluated quantitatively using a Bioimager system (BD Biosciences Rockville, Montgomery, MD, USA) and the fluorescence intensity was measured at 485/530 nm. 

### 2.5. Statistical Significance

The experimental data were expressed as the mean ± standard deviation of one or more individual experiments wherever applicable. The analysis of experimental data was performed with the students t-test using Graph Pad Prism 6.0 (San Diego, CA, USA) and statistically significant values were indicated as *p* < 0.05. 

## 3. Results and Discussion

### 3.1. Dynamic Light Scattering

**Figure 1:**
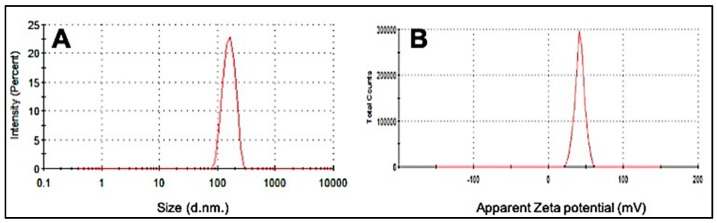
Dynamic light scattering (DLS) images of (**A**) average particle size distribution of CS Nanoparticle and (**B**) Zeta potential of synthesized CS Nanoparticle.

Particle size and surface charge are two important factors determining the size, stability, and effective delivery of the drug to the target site [24]. Figure 1A depicts the average particle size distribution of the synthesized CS Np using an ionotropic gelation method. As can be observed, the bare nanoparticles have an average size of 197.8 ± 49.18 nm with a PDI 0.244. 

The zeta potential is a measure of the stability and surface charge on the particles. A high zeta potential is indicative of high electric surface charge allowing strong repulsion between the surrounding particles and hence preventing aggregation [24]. Figure 1B depicts the average zeta potential value of CS Np which was observed to be +36.0 ± 4.68. A previous study on chitosan nanoparticles showed that the blank nanoparticles in the ratio 5:1 CS/TPP had a particle size in the range of 300 to 390 nm and a zeta potential of + 44 ± 5.2, which strongly supports our findings [25].

### 3.2. Morphological Characterization

**Figure 2:**
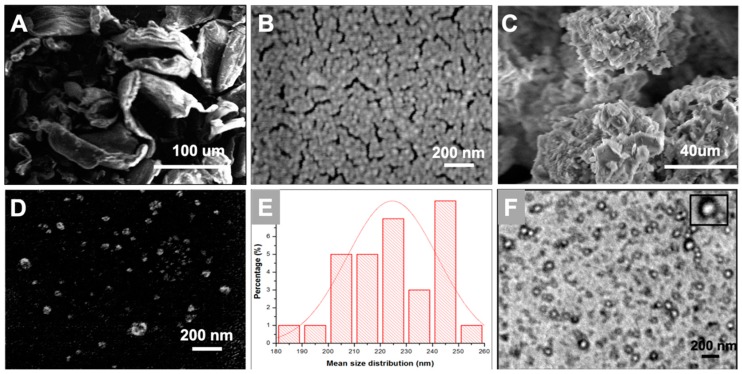
Scanning Electron Microscopy images of (**A**) chitosan powder; (**B**) freshly prepared CS Np and (**C**,**D**) lyophilized CS Np. Further, (**E)** depicts the average size distribution of the nanoparticles obtained from scanning electron microscopy. Whereas, (**F**) depicts the transmission electron microscopic image of chitosan nanoparticles.

Figure 2 displays the scanning electron micrograph images of chitosan powder, freshly prepared nanoparticles, and lyophilized chitosan nanoparticles sample. It was observed that the nanoparticles displayed spherically compact structures with an average diameter of 220 ± 40 nm, see Figure 2E, which almost coincides with our dynamic light scattering study. Similar results were reported in another study where the CS/TPP in a 5:1 ratio displayed an average size of 200 ± 24 nm [26,27]. The freshly prepared CS Np appeared as clusters. This can be attributed to the fusion of particles through hydrogen bonding [24]. Moreover, the stability of chitosan nanoparticles was tested in buffer solution (pH 7.4). The morphology of the nanoparticles was observed to be spherical with an average diameter of 200 nm, see Supplementary Figure S1.

**Figure 3:**
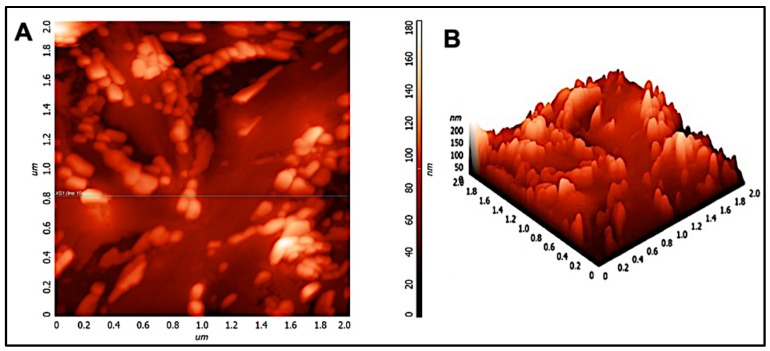
(**A**) 2D and (**B**) 3D atomic force micrograph of chitosan nanoparticles in a 2-micron × 2-micron area.

Transmission electron microscopy is a technique which provides information on the morphology and size of the particles. Figure 3 depicts a TEM micrograph of small and spherical chitosan nanoparticles with a diameter of around 120 ± 30 nm. The size of the nanoparticles in the TEM image, see Figure 2F, are smaller than that represented by SEM and DLS. This can be attributed to the aggregation of nanoparticles due to their high surface area and energy which generates a larger entity [28]. However, dimensions of freshly prepared single particles can be observed clearly in the TEM image of the CS Np sample.

### 3.3. AFM Analysis

Scanning electron microscopy and transmission electron microscopy provides a two-dimensional projection or image of the nanoparticles. However, atomic force microscopy is a powerful technique which enables a three-dimensional surface profile of the nanoparticles to be viewed. In addition, it can also provide accurate heights of the nanoparticles. Figure 3 shows the atomic force micrograph of chitosan nanoparticles. As can be observed, the nanoparticles appeared to be spherical in shape. Furthermore, the size distribution histogram was performed for the nano-chitosan in a 2-micron × 2-micron area. Analysis shows that the average particle size is around 200 nm as the histogram peaks are at 0.2 microns, see Supplementary Figure S2, which coincides with our DLS, SEM, and TEM data. Similar results were observed in previous reports [29,30,31]. In addition, a small peak appeared at 600 nm which can be attributed to the fusion of particles through hydrogen bonding [24]. 

### 3.4. Spectroscopic Characterization

**Figure 4:**
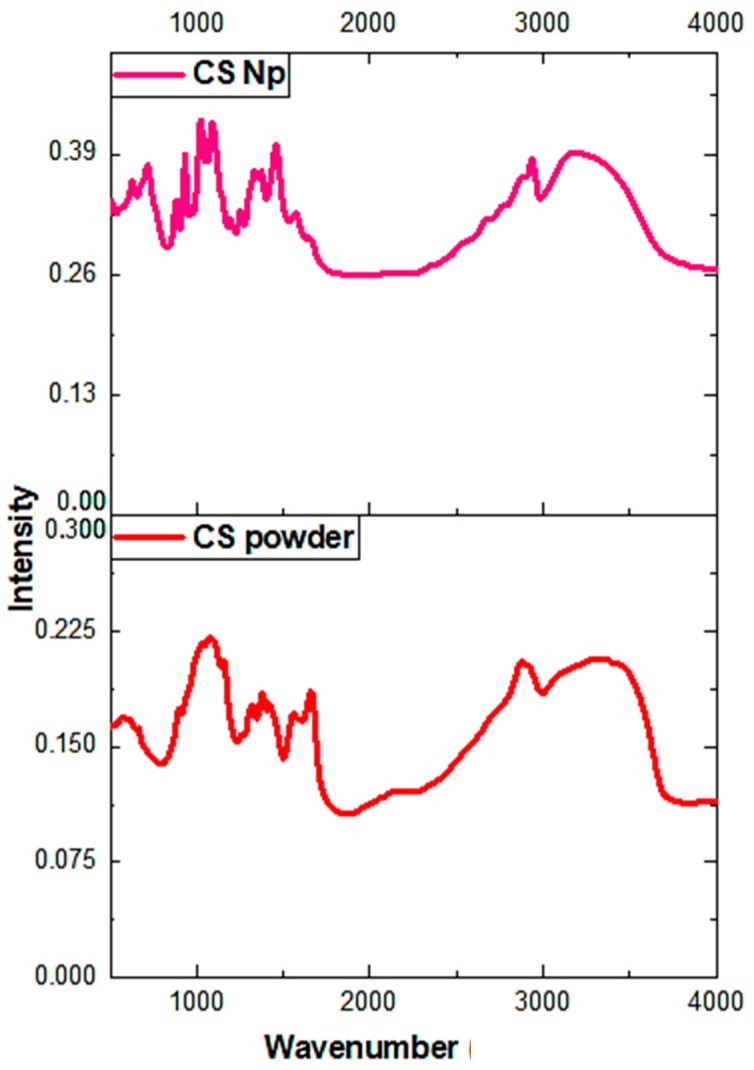
Fourier-transform infrared spectroscopy (FTIR) spectra of chitosan nanoparticles and chitosan powder.

Fourier-transform infrared spectroscopy (FTIR) is a powerful spectroscopic technique to determine the chemical composition and presence of a drug inside the nanoparticles. Figure 4 represents the FTIR spectra of chitosan powder and chitosan nanoparticles. In the FTIR spectrum of chitosan nanoparticles, a broad peak at 3436 cm^−1^ corresponds to the -NH and -OH stretching vibrations. The weak band at 2930 cm^−1^ corresponds to –CH stretching, whereas vibrational bands at 1640 cm^−1^, 1560 cm^−1^, and 1320 cm^−1^ may be attributed to the amide carbonyl stretch and -NH bend of the amine groups in the chitosan nanoparticles [32]. The band at 1099 cm^−1^ represents C–O bond stretching. Moreover, the vibrational band at 860 cm^−1^ corresponds to the CH_2_OH group in the pyranose ring of chitosan. The only difference between the spectra of chitosan powder and chitosan nanoparticles occurred at 1155 cm^−1^, which could be assigned to the linkage between phosphate groups of the sodium tripolyphosphate and ammonium ions of the chitosan group. Furthermore, our results agree with report of Gopalakrishnan et al. (2014) and Jafary et al. (2016) [24,32].

### 3.5. Effect of Chitosan Nanoparticles on Rotenone-Induced Cell Death

**Figure 5:**
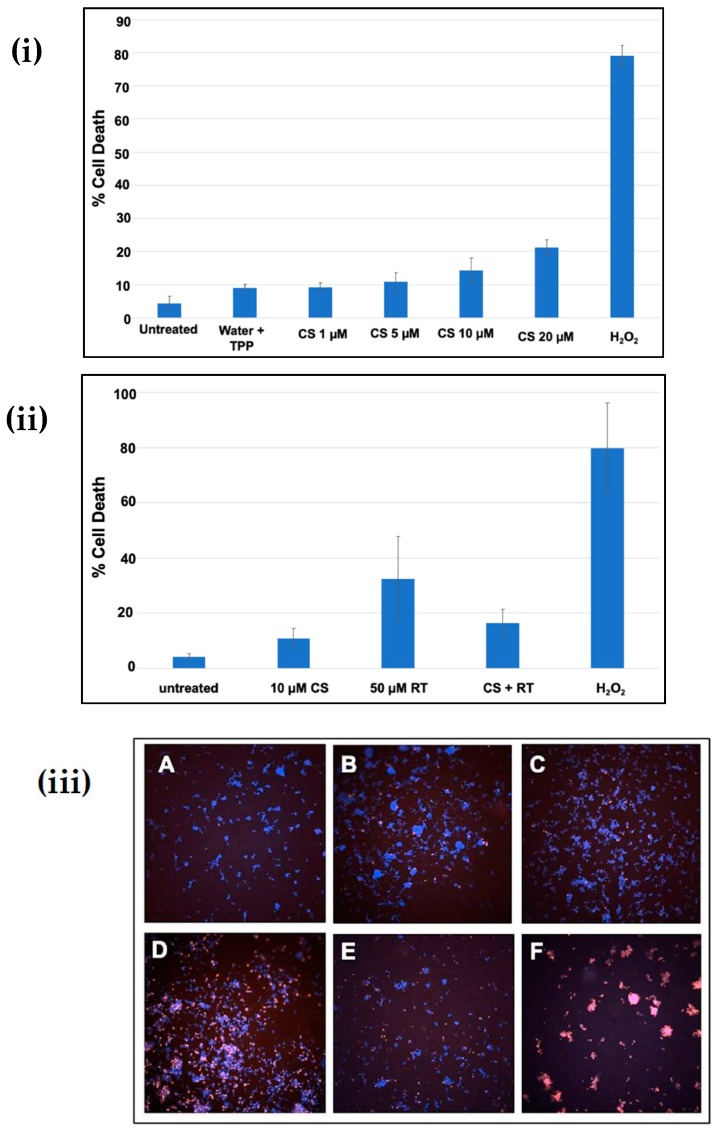

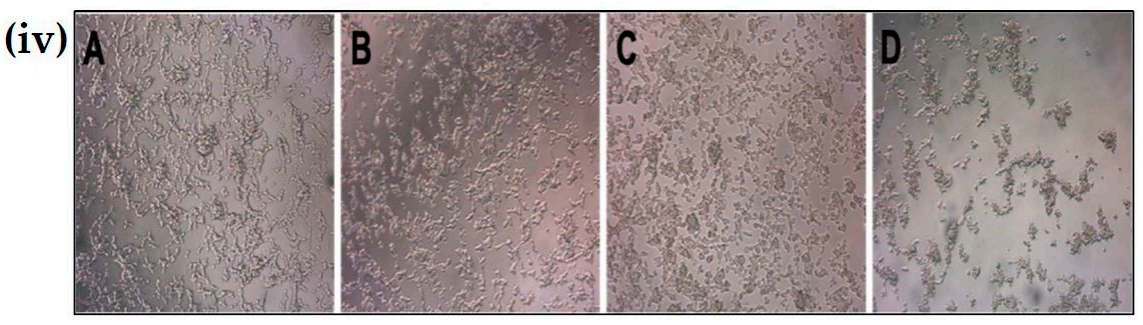
(**i**) Cytotoxicity of chitosan nanoparticles at different concentrations after 24 h of incubation; (**ii**) Protective effect of chitosan nanoparticles against rotenone insult in SH-SY5Y cells; (**iii**) Hoechst-propidium iodide staining images of (**a**) untreated cells, (**b**) water + Tripoly phosphate; (**c**) chitosan nanoparticles 10 µM concentration, (**d**) 50 µM rotenone, (**e**) CS Np + Rotenone and (**f**) hydrogen-peroxide-treated cells after 24 h of treatment; (**iv**) Bright field microscopy images of (**a**) untreated cells, (**b**) CS Np 10 µM, (**c**) CS Np + RT, & (**d**) 50 µM RT cells visualized using a compound microscope after 24 h of treatment. Each experimental point was assessed in quintuplicate.

The cytotoxicity of chitosan nanoparticles and its protective function against rotenone insult in SH-SY5Y cell line were checked using a high-throughput screening assay. Figure 5i shows cells exposed to an increasing concentration (1–20 µM) of chitosan nanoparticles exhibited cytotoxicity from 9% to 21%. A 10 µM concentration did not display much difference compared to the untreated and vehicle controls. However, on addition of 50 µM rotenone, around 35–50% of the cell death was reported in the SH-SY5Y cell line after 24 h of incubation time, see Figure 5ii. In contrast, the addition of a 10 µM chitosan nanoparticle solution prior to rotenone exposure resulted in 14–20% cell death compared to 35–50% cell death upon rotenone administration. These data show that treatment of cells with chitosan prior to rotenone exposure attenuated cell death by 25–30%. The morphology of cells after rotenone treatment and cells treated with chitosan prior to rotenone exposure further displays the protective aspect of chitosan nanoparticles against rotenone insult. Bright field microscopy images and Hoechst 33342-propidium iodide staining pictures, see Figure 5iii, further support the protective aspect of chitosan nanoparticles. The pretreatment of cells with a 10 µM chitosan nanoparticles solution for 6 h enabled the cells to retain their cellular morphology, as depicted by bright field microscopy images. In contrast, cells treated with 50 µM rotenone showed a morphology similar to that of cells exposed to 50 µM hydrogen peroxide, see Figure 5iv. Hydrogen peroxide was used as a positive control at a concentration of 50 µM. 

### 3.6. Flow Cytometry Analysis

**Figure 6:**
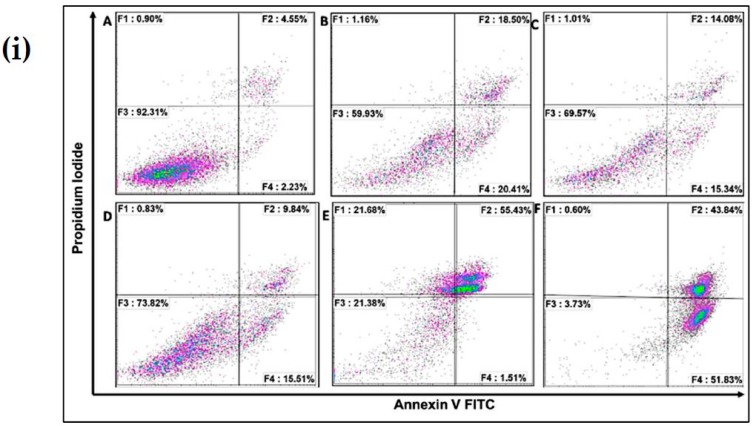

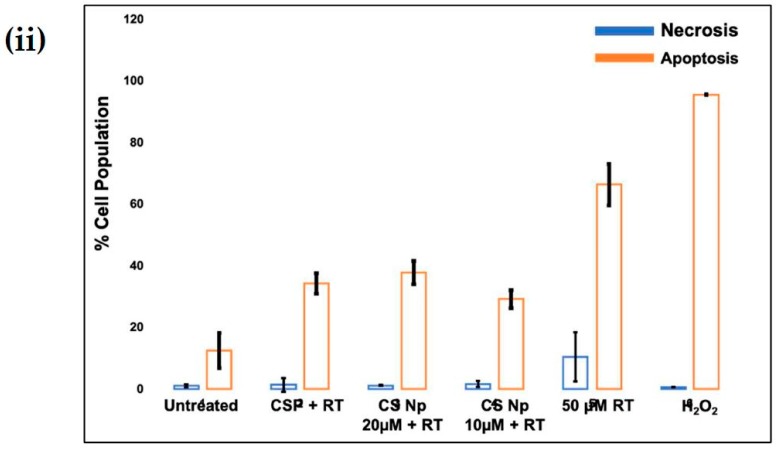
(**i**) Flow cytometry matrix plots used to measure apoptosis/necrotic distribution for (**A**) untreated, (**B**) 20 µM Chitosan powder + RT, (**C**) CS Np 20 µM, (**D**) CS Np+ RT, (**E**) 50 µM RT, and (F) hydrogen peroxide after 24 h of treatment; (**ii**) quantification of apoptotic/necrotic assay under the previously mentioned conditions. Each experimental point was assessed in triplicate.

The mechanism by which cell-death occurs (apoptotic pathway or necrotic pathway) was examined. Figure 6i,ii shows cells pre-treated with 20 µM chitosan powder, 10 µM chitosan nanoparticles, and 20 µM chitosan nanoparticles. A concentration of 50 µM hydrogen peroxide was used as a positive control whereas untreated cells were used as a negative control. As can be observed, cells treated with 50 µM rotenone showed a substantial increase in early and late apoptosis (lower right and top right quadrants of the plot) 24 h after administration. A similar result was observed when cells were treated with 50 µM hydrogen peroxide. However, prior treatment of cells with 20 µM chitosan powder and 10 µM and 20 µM chitosan nanoparticles resulted in a notable rescue of the cells from rotenone insult (apoptotic-cell death). A notable difference was observed when 50 µM rotenone and CS Np 10 µM + rotenone treatments were compared. Thus, we can conclude that chitosan nanoparticles exhibit anti-apoptotic activity. 

### 3.7. Chitosan Np Prevents Rotenone-Induced Mitochondrial Dysfunction 

**Figure 7:**
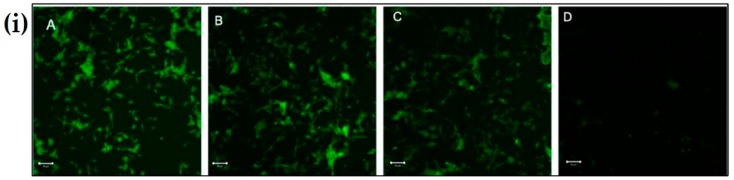

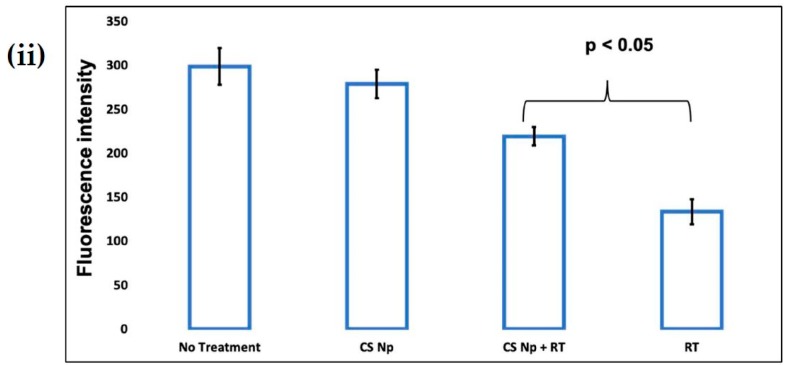
The effect of CS Np on rotenone insult determined using rhodamine 123 dye. (**i**) Cells were incubated with rhodamine 123 and the mitochondrial membrane potential was monitored using a fluorescent microscope for (**A**) untreated, (**B**) CS Np 20 µM, (**C**) 20 µM Chitosan powder + RT, and (**D**) 50 µM RT; (**ii**) quantification of Mitochondrial membrane potential assay under previously described conditions. Each experimental point was assessed in triplicate. The scale bar in the image is 50 µm.

The untreated cells, see Figure 7(iA), and cells treated with chitosan nanoparticles, see Figure 7(iB), exhibited green fluorescence, indicating that a large fraction of mitochondria inside the cell were in an energized state. However, cells upon treatment with rotenone, see Figure 7(iD), displayed decreased mitochondrial energy transduction as observed by the disappearance of green fluorescence. Further, cells treated with chitosan nanoparticles prior to rotenone treatment, see Figure 7(iC), exhibited green fluorescence which can be attributed to the inhibition of the collapse of the membrane potential via rescue of mitochondrial membrane depolarization by chitosan nanoparticles. Thus, these results suggest that chitosan nanoparticles may inhibit rotenone-induced cellular apoptosis through a mitochondria-involved pathway, as revealed by the considerable increase in green fluorescence intensity.

## 4. Proposed mechanism

**Figure 8:**
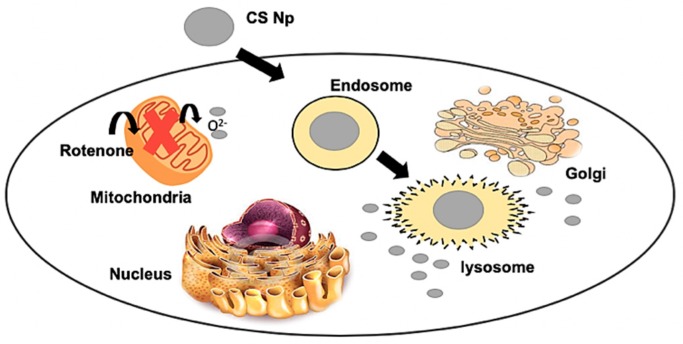
Mechanistic model of the action of chitosan nanoparticles.

We henceforth propose a mechanistic model for the action of chitosan nanoparticles (Figure 8). Firstly, chitosan nanoparticles are internalized by the cells through endocytosis via an endosome–lysosome pathway. Later, these nanoparticles are released into the cytoplasm from the lysosome due to the low pH environment and enzymatic activity. These nanoparticles then scavenge the reactive oxygen species generated by the inhibition of complex I of the ETC of mitochondria by the rotenone insult. Thus, the apoptotic stimuli exerted on mitochondria is reduced. Subsequently, cytochrome c cannot be released through the permeability transition pore. Further, caspase 3 cannot be activated by caspase 9, resulting in inhibition of Poly ADP ribose polymerase (PARP) cleavage and hence, cell survival [22,23]. Therefore, chitosan nanoparticles protect the cell from rotenone insult due to its anti-oxidant and anti-apoptotic activity. 

## 5. Conclusions

The literature is flooded with various number of reports suggesting that oxidative stress and consequent cellular apoptosis are important factors in rotenone-induced cell death. Therefore, pre-treatment of cells with antioxidants such as low molecular weight sulphonated chitosan, fucoidan, quercetin, flavonoids, etc., likely reduces the adverse effects arising from rotenone insult. Although pre-treatment of cells with the above mentioned anti-oxidants were observed to alleviate reactive oxygen species production, the antioxidant effects of chitosan nanoparticulate system against rotenone insult had not been studied previously. In this regard, our study demonstrates the synthesis of chitosan nanoparticles by an ionic gelation method using TPP as the cross-linking agent. The optimized ratio of CS/TPP was 5:1, which produced spherical nanoparticles with an average size of 200 nm as observed by SEM, TEM, DLS, and AFM. Further, the cytotoxicity of the chitosan nanoparticles and its anti-oxidant and anti-apoptotic effects against rotenone-induced cell death were determined using a differential nuclear staining cytotoxicity assay and flow cytometric analysis. Therefore, Chitosan Nanoparticles could inhibit the rotenone-induced mitochondria involved apoptosis pathway, as revealed by the considerable increase in the green fluorescence intensity in the MMP assay. These findings suggest that chitosan nanoparticles might be a useful and promising neuroprotective agent for the prevention of Parkinson’s disease. 

## Supplementary Materials

The following are available online at https://www.mdpi.com/1996-1944/12/7/1176/s1, Figure S1: SEM image of Chitosan nanoparticles in buffer solution, Figure S2: Size distribution analysis from AFM measurement.
