# Supplementary material for: Chitosan Nanoparticles Rescue Rotenone-Mediated Cell Death
Source: Materials (Basel). 2019 Apr 11;12(7):1176. doi: 10.3390/ma12071176 (PMC6480189; doi:10.3390/ma12071176)
Supplement: Supplementary file 1 [file materials-12-01176-s001.pdf]

materials

Supplementary Information

Chitosan Nanoparticles Rescue Rotenone-Mediated Cell Death

Jyoti Ahlawat [1], Eva M Deemer [2] and Mahesh Narayan [1,*]

1 Department of Chemistry & Biochemistry; The University of Texas at El Paso; Texas 79968; USA; jahlawat@miners.utep.edu
2 Material Science & Engineering department; The University of Texas at El Paso; Texas 79968; USA; emdeemer@utep.edu
* Correspondence: mnarayan@utep.edu; Tel.: +1 (915)-747-6614; Fax: +1 (915)-7478383

Received: 13 March 2019; Accepted: 4 April 2019; Published: 11 April 2019

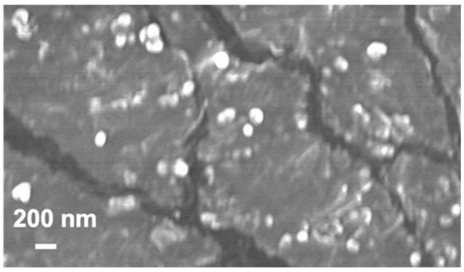

Supplementary Figure 1. SEM image of Chitosan nanoparticles in buffer solution.

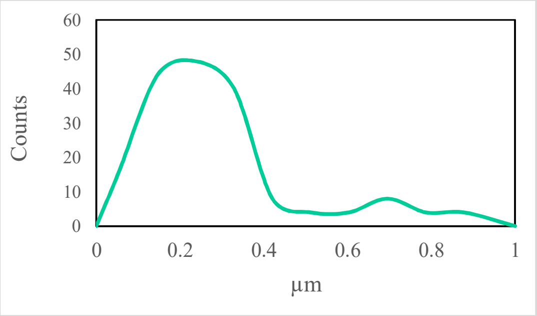

Supplementary Figure 2. Size distribution analysis from AFM measurement.
